# Chaos Enhanced Differential Evolution in the Task of Evolutionary Control of Selected Set of Discrete Chaotic Systems

**DOI:** 10.1155/2014/836484

**Published:** 2014-08-26

**Authors:** Roman Senkerik, Ivan Zelinka, Michal Pluhacek, Donald Davendra, Zuzana Oplatková Kominkova

**Affiliations:** ^1^Faculty of Applied Informatics, Tomas Bata University in Zlin, Náměstí T.G. Masaryka 5555, 760 01 Zlin, Czech Republic; ^2^Faculty of Electrical Engineering and Computer Science, VŠB-Technical University of Ostrava, 17 Listopadu 15, Poruba, 708 33 Ostrava, Czech Republic

## Abstract

Evolutionary technique differential evolution (DE) is used for the evolutionary tuning of controller parameters for the stabilization of set of different chaotic systems. The novelty of the approach is that the selected controlled discrete dissipative chaotic system is used also as the chaotic pseudorandom number generator to drive the mutation and crossover process in the DE. The idea was to utilize the hidden chaotic dynamics in pseudorandom sequences given by chaotic map to help differential evolution algorithm search for the best controller settings for the very same chaotic system. The optimizations were performed for three different chaotic systems, two types of case studies and developed cost functions.

## 1. Introduction

In many engineering applications, one of the most innate tasks is the controlling of highly nonlinear dynamical systems in order to either eliminate or synchronize the chaos. The first pioneering approach to control chaotic dynamics by means of a simple analytical linearization method was introduced in 1990s by Ott et al. (i.e., OGY method) [1]. Subsequently, the rapid development of methods for stabilizing of nonlinear chaotic dynamics has arisen and many advanced techniques have been applied for chaos control and chaos synchronization including methods from the artificial intelligence field.

During recent years, usage of new intelligent systems in engineering, technology, modeling, computing, and simulations has attracted the attention of researchers worldwide. The most current methods are mostly based on soft computing, which is a discipline tightly bound to computers, representing a set of methods of special algorithms, belonging to the artificial intelligence paradigm. The most popular of these methods are neural networks, evolutionary algorithms (EAs), fuzzy logic, and tools for symbolic regression like genetic programming. Currently, EAs are known as a powerful set of tools for almost any difficult and complex optimization problem.

The interest about the interconnection between evolutionary techniques and control of chaotic systems is rapidly spreading. The initial research was conducted in [2], whereas [3, 4] was more concerned with the tuning of parameters inside the chaos control technique based on the Pyragas method: extended delay feedback control (ETDAS) [5]. When compared to the aforementioned research, later works [6, 7] show a possibility as to how to generate the entire control law (not only how to optimize several parameters) for the purpose of stabilization of a chaotic system. Such approach also may overcome the possible sensitivity to initial conditions which may lead to stability issues. The synthesis of control is inspired by the Pyragas' delayed feedback control technique [8, 9]. This method is very advantageous for the evolutionary computation, due to its amount of easy accessible control parameters, which can be easily tuned by means of EAs.

Other approaches utilizing the EAs for stabilizing chaotic dynamics have mostly applied the particle swarm optimization (PSO) algorithm [10] and multi-interval gradient method [11] or minimum entropy control technique [12]. EAs have been also frequently used in the task of synchronization of chaos [13–15]. In [16], an EA for optimizing local control of chaos based on a Lyapunov approach is presented.

Evolutionary techniques were also used for the synthesis of new complex discrete type structures with chaotic behavior [17] as well as the synthesis (identification) of a mathematical model of chaotic system based on the measured data [18].

Another example of interconnection between deterministic chaos and EAs represents the research focused on the embedding of chaotic dynamics into the EAs. Recent research in chaos driven heuristics has been fueled with the predisposition that, unlike stochastic approaches, a chaotic approach is able to bypass local optima stagnation. This one clause is of deep importance to EAs. A chaotic approach generally uses the chaotic map in the place of a pseudorandom number generator [19]. This causes the heuristic to map unique regions, since the chaotic map iterates to new regions. The task is then to select a very good chaotic map as the pseudorandom number generator.

The initial concept of embedding chaotic dynamics into EAs is given in [20]. Later, the initial study [21] was focused on the simple embedding of chaotic systems in the form of chaos pseudorandom number generator (CPRNG) for differential evolution (DE) [22] and self-organizing migrating algorithm (SOMA) [23] in the task of optimal PID tuning. Also, the PSO algorithm with elements of chaos was introduced as the CPSO [24]. This field of research was later extended with the successful experiments with chaos driven DE [25] in real domain as well as in combinatorial problems domain [26, 27].

At the same time, the chaos embedded PSO with inertia weigh strategy was closely investigated [28], followed by the introduction of a PSO strategy driven alternately by two chaotic systems [29] and novel chaotic multiple choice PSO (chaos MC-PSO) strategy [30]. Recently, the chaotic firefly algorithm was also introduced [31].

Finally, the last example represents the research focusing on the EAs and the edge of chaos. An unconventional approach of the edge of chaos and its application to discrete systems and evolutionary algorithms in terms of stagnation avoidance is presented in [32].

The organization of this paper is as follows: firstly, used evolutionary technique, which is DE, is described and is followed by the description of the ChaosDE concept. Thereafter, the problem design and appropriate corresponding cost functions are investigated and proposed. Results and conclusion follow afterwards.

## 2. Motivation

This paper extends the research of evolutionary chaos control optimization by means of ChaosDE algorithm [33–35]. Recent studies have shown that differential evolution [22] is one of the most potent heuristics and it has been used for a number of optimization tasks; [36–38] have explored DE for combinatorial problems; [39, 40] have hybridized DE whereas [41–43] have developed self-adaptive DE variants.

In this paper, the DE/rand/1/bin strategy driven by different chaotic maps (systems) was utilized to solve the issue of evolutionary optimization of chaos control for the very same chaotic system used as a CPRNG in the particular case study. Thus, the idea was to utilize the hidden chaotic dynamics in pseudorandom sequences given by chaotic map to help differential evolution algorithm search for the best controller settings for the very same chaotic system. Since the very positive contribution of the chaotic dynamics to the performance of DE in the task of evolutionary chaos control optimization was proven in comparison with original canonical DE within the initial study [44], this paper is not primarily focused on the performance comparisons with different heuristic. But this research extends the initial work with the aforementioned idea and with the several case studies combining different chaotic systems and different utilized cost functions.

## 3. Used Heuristic: Differential Evolution (DE)

DE is a simple and powerful population-based optimization method that works either on real-number-coded individuals or with small modifications on discrete type individuals [22, 45, 46]. DE is quite robust, fast, and effective, with global optimization ability. This global optimization ability has been proven in many interdisciplinary researches. It works well even with noisy and time-dependent objective functions. The canonical basic principle is as follows.

For each individual ${\vec{x}}_{i,G}$ in the current generation G, DE generates a new trial individual ${\vec{x}}_{i,G}^{\prime }$ by adding the weighted difference between two randomly selected individuals ${\vec{x}}_{r1,G}$ and ${\vec{x}}_{r2,G}$ to a randomly selected third individual ${\vec{x}}_{r3,G}$. The resulting individual ${\vec{x}}_{i,G}^{\prime }$ is crossed over with the original individual ${\vec{x}}_{i,G}$. The fitness of the resulting individual, referred to as a perturbed vector ${\vec{u}}_{i,G+1}$, is then compared with the fitness of ${\vec{x}}_{i,G}$. If the fitness of ${\vec{u}}_{i,G+1}$ is greater than the fitness of ${\vec{x}}_{i,G}$, then ${\vec{x}}_{i,G}$ is replaced with ${\vec{u}}_{i,G+1}$; otherwise, ${\vec{x}}_{i,G}$ remains in the population as ${\vec{x}}_{i,G+1}$.

Please refer to (1) for notation of crossover and to [22] for the detailed description of used DERand1Bin strategy and all other DE strategies:

(1) ui,G+1=xr1,G+F·(xr2,G−xr3,G).

## 4. Concept of ChaosDE

This section contains the description of discrete dissipative chaotic maps, which can be used as the chaotic pseudorandom generators for DE as well as the main principle of the ChaosDE concept. In this research, direct output iterations of the chaotic maps were used for the generation of real numbers in the process of crossover based on the user defined* CR* value and for the generation of the integer values used for selection of individuals.

### 4.1. Chaotic Pseudorandom Number Generator

The general idea of ChaosDE and CPRNG is to replace the default PRNG with the discrete chaotic map. As the discrete chaotic map is a set of equations with a static start position, we created a random start position of the map, in order to have different start position for different experiments (runs of EAs). This random position is initialized with the default PRNG, as a one-off randomizer. Once the start position of the chaotic map has been obtained, the map generates the next sequence using its current position.

The first possible way is to generate and store a long data sequence (approximately 50–500 thousands numbers) during the evolutionary process initialization and keep the pointer to the actual used value in the memory. In case of the using up of the whole sequence, the new one will be generated with the last known value as the new initial one.

The second approach is that the chaotic map is not reinitialized during the experiment and any long data series is not stored; thus it is imperative to keep the current state of the map in memory to obtain the new output values.

As two different types of numbers are required in ChaosDE, real and integers, the use of modulo operators is used to obtain values between the specified ranges, as given in the following equations (2):

(2) $$\begin{array}{c} \text{rndreal}=\mathrm{mod}\left(\text{abs}\left(\text{rndChaos}\right),1.0\right)\;\\ \text{rndint}=\mathrm{mod}\left(\text{abs}\left(\text{rndChaos}\right),1.0\right)\times \text{Range}+1, \end{array}$$

where abs refers to the absolute portion of the chaotic map generated number rndChaos, mod is the modulo operator, and Range specifies the value (inclusive) till where the number is to be scaled.

### 4.2. Selected Chaotic Systems

This subsection contains the mathematical and graphical description of the three selected discrete dissipative systems, which served both as for CPRNGs and also as the examples of systems to be evolutionary controlled.

#### 4.2.1. Burgers Map

The Burgers mapping is a discretization of a pair of coupled differential equations which were used by Burgers to illustrate the relevance of the concept of bifurcation to the study of hydrodynamics flows. The map equations are given in (3) with control parameters *a* = 0.75 and *b* = 1.75 as suggested in [47]:

(3) $$\begin{array}{c} X_{n+1}=aX_{n}-Y_{n}^{2},\\ Y_{n+1}=bY_{n}+X_{n}Y_{n}. \end{array}$$

#### 4.2.2. Delayed Logistic

The delayed logistic is a simple two-dimensional discrete system similar to the one-dimensional logistic equation. The map equations are given in (4). The parameter used in this work is *A* = 2.27 as also suggested in [47]:

(4) $$\begin{array}{c} X_{n+1}=AX_{n}\left(1-Y_{n}\right),\\ Y_{n+1}=X_{n}. \end{array}$$

#### 4.2.3. Lozi Map

The Lozi map is a simple discrete two-dimensional chaotic map. The map equations are given in (5). The parameters used in this work are as follows: *a* = 1.7 and *b* = 0.5 as suggested in [47]. For these values, the system exhibits typical chaotic behavior and, with this parameter setting, it is used in most research papers and other literature sources [48]:

(5) $$\begin{array}{c} X_{n+1}=1-a\left|X_{n}\right|+bY_{n},\\ Y_{n+1}=X_{n}. \end{array}$$

### 4.3. Graphical Examples

**Figure 1:**
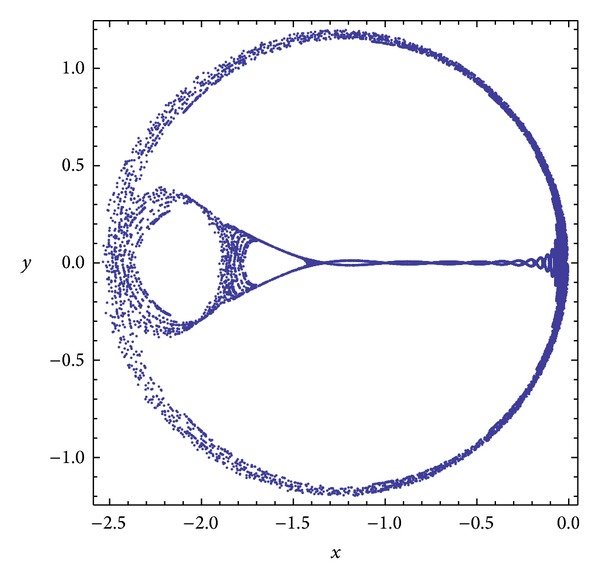
*x*, *y* plot of the Burgers map.

**Figure 2:**
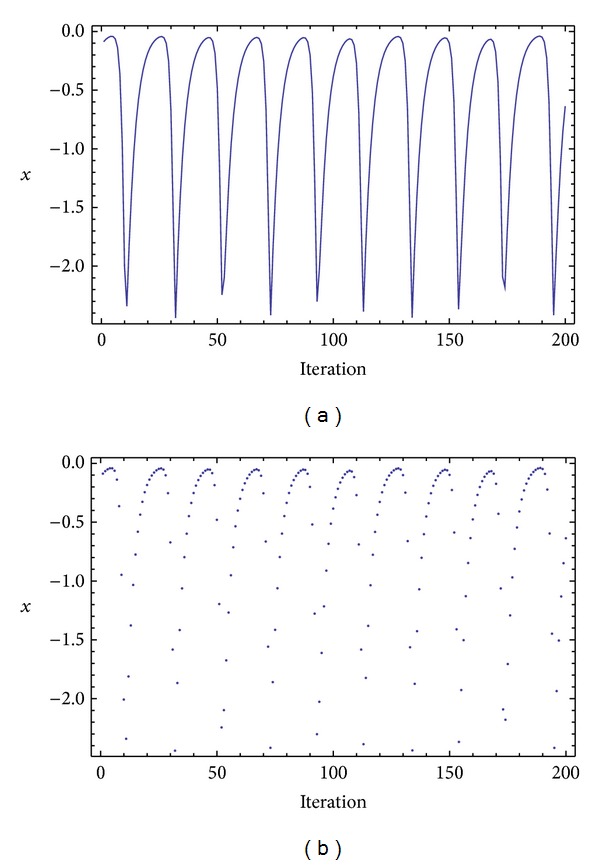
Iterations of the Burgers map (variable *x*) (a), iterations of the Burgers map (variable *x*), point-plot (b).

**Figure 3:**
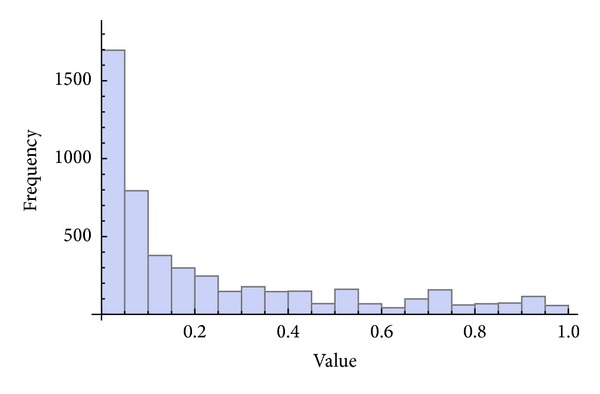
Histogram of the distribution of real numbers transferred into the range 〈0-1〉 generated by means of the chaotic Burgers map, 5000 samples.

**Figure 4:**
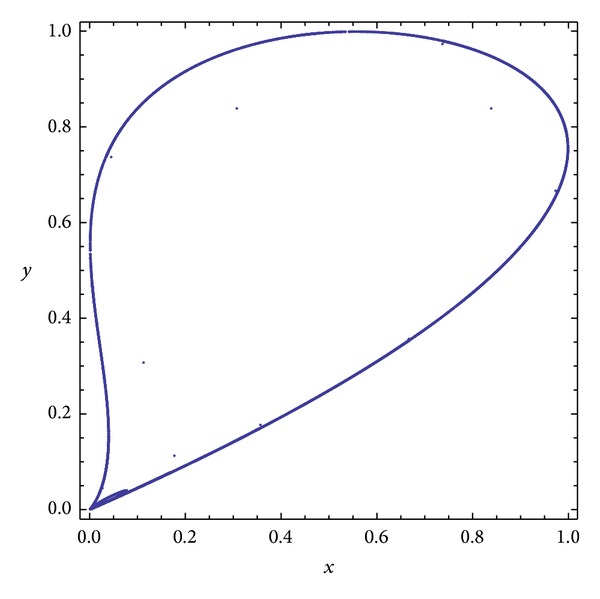
*x*, *y* plot of the delayed logistic map.

**Figure 5:**
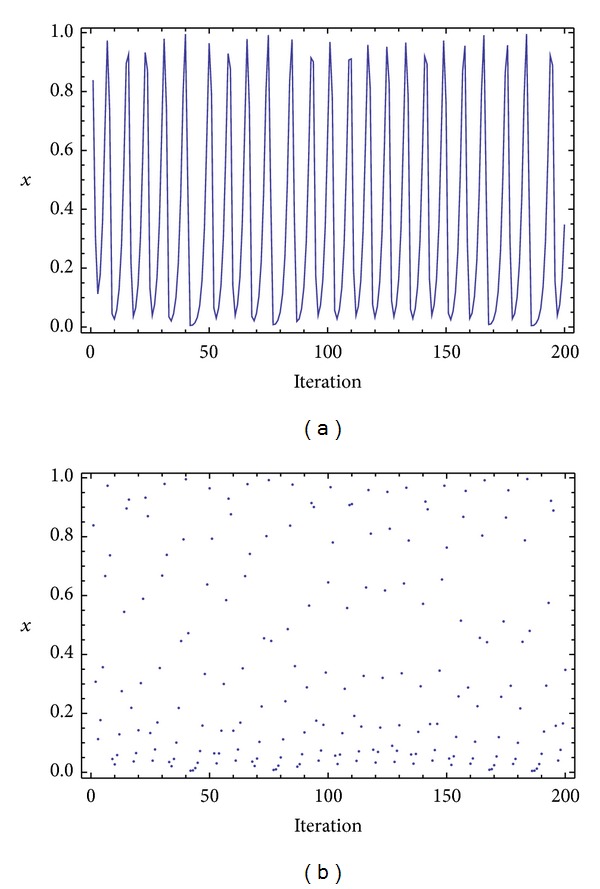
Iterations of the delayed logistic (variable *x*) (a), iterations of the delayed logistic (variable *x*), point-plot (b).

**Figure 6:**
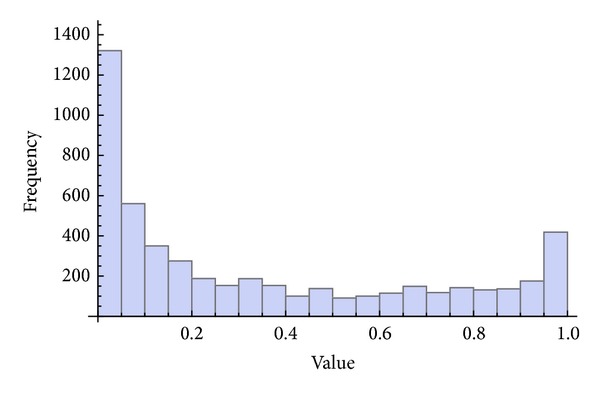
Histogram of the distribution of real numbers transferred into the range 〈0-1〉 generated by means of the chaotic delayed logistic map, 5000 samples.

**Figure 7:**
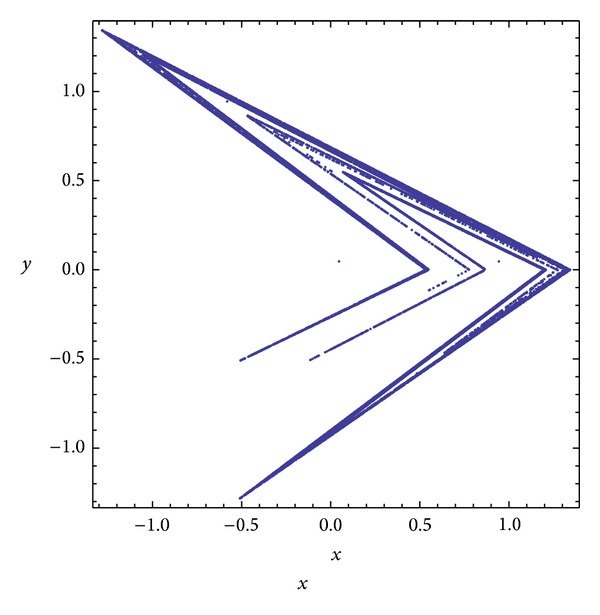
*x*, *y* plot of the Lozi map.

**Figure 8:**
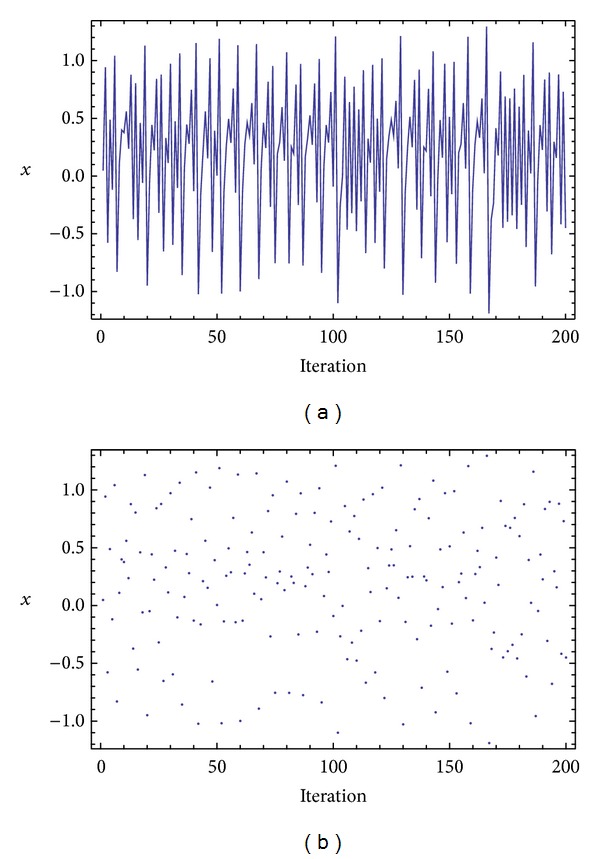
Iterations of the Lozi map (variable *x*) (a), iterations of the Lozi map (variable *x*), point-plot (b).

**Figure 9:**
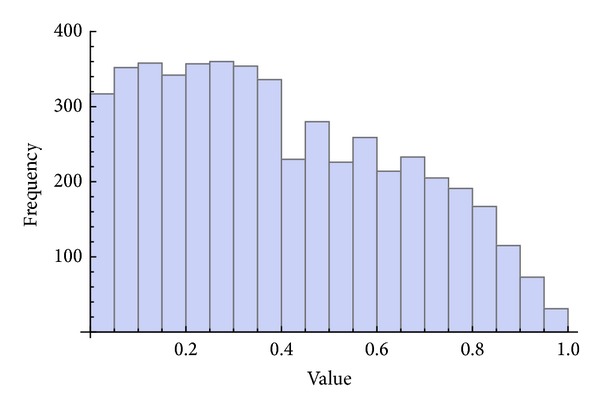
Histogram of the distribution of real numbers transferred into the range 〈0-1〉 generated by means of the chaotic Lozi map, 5000 samples.

Three chaotic maps were selected for the CPRNG concept. The *x*, *y* plots of the selected maps are depicted in Figure 1 (Burgers map), Figure 4 (delayed logistic map), and Figure 7 (Lozi map). The chaotic behavior of the chaotic maps, represented by the examples of direct output iterations, is depicted in Figures 2, 5, and 8. Finally, the illustrative histograms of the distribution of real numbers transferred into the range 〈0-1〉 generated by means of chaotic maps are in Figures 3, 6, and 9.

## 5. Design of Cost Functions for Chaotic System Stabilization

The proposal of the basic cost function (CF_Simple_) is in general based on the simplest CF, which could be used problem-free only for the stabilization of p-1 orbit. The idea was to minimize the area created by the difference between the required state and the real system output on the whole simulation interval, *τ*_*i*_ (6). This CF design is very convenient for the evolutionary searching process due to the relatively favorable CF surface. Nevertheless, this simple approach has one big disadvantage, which is the including of initial chaotic transient behavior of not stabilized system into the cost function value. As a result of this, the very tiny change of control method setting for extremely sensitive chaotic system (given by the very small change of CF value) can be suppressed by the aforementioned inclusion of initial chaotic transient behavior. Consider

(6) $$\begin{array}{c} {\text{CF}}_{\text{Simple}}=\overset{\tau_{i}}{\underset{t=0}{\sum }}\left|{\text{TS}}_{t}-{\text{AS}}_{t}\right|, \end{array}$$

where TS is target state and AS is actual state.

**Figure 10:**
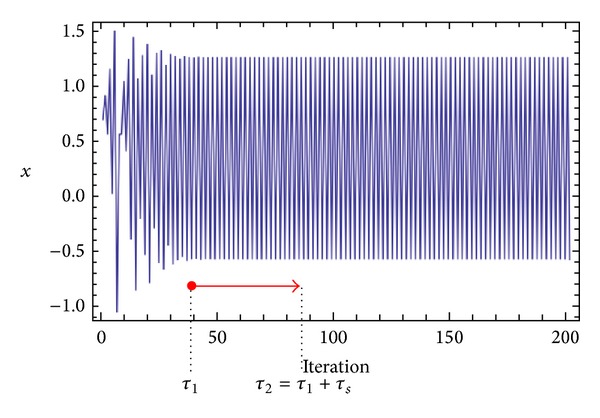
“Floating window” for minimization.

Different type of universal cost function without any selection rules is purely based on searching for the desired stabilized periodic orbit and thereafter calculation of the difference between desired and found actual periodic orbit on the short time interval, *τ*_*s*_ (20 iterations), from the point where the first minimal value of difference between desired and actual system output is found (i.e., floating window for minimization; see Figure 10).

Such a design of universal CF should secure the successful stabilization of either p-1 orbit (stable state) or any higher periodic orbit anywise phase shifted. Furthermore, due to CF values converging towards zero, this CF also allows the use of decision rules, avoiding very time demanding simulations. This rule stops EA immediately, when the first individual with good parameter structure is reached; thus the value of CF is lower than the acceptable (CF_acc_) one. Based on the numerous experiments, typically CF_acc_ = 0.001 at time interval *τ*_*s*_ = 20 iterations; thus the difference between desired and actual output has the value of 0.0005 per iteration, that is, successful stabilization for the used control technique. The CF_UNI_ has the following form:

(7) $$\begin{array}{c} \text{C}{\text{F}}_{\text{UNI}}=pen_{1}+\overset{\tau 2}{\underset{t=\tau 1}{\sum }}\left|{\text{TS}}_{t}-{\text{AS}}_{t}\right|, \end{array}$$

where *τ*_1_ is the first min value of the difference between TS and AS and *τ*_2_ is the end of optimization interval (*τ*_1_ + *τ*_*s*_), *pen*_1_ = 0 if *τ*_*i*_ − *τ*_2_ ≥ *τ*_*s*_; *pen*_1_ = 10∗(*τ*_*i*_ − *τ*_2_) if *τ*_*i*_ − *τ*_2_ < *τ*_*s*_ (i.e., late stabilization).

The issue of pure searching for periodic orbits causes very chaotic, erratic, and discrete type CF surfaces.

## 6. Experimental Design

This research encompasses six case studies. Six different sets of discrete chaotic systems as CPRNGs/to be controlled and two different cost functions were combined in the following form:

- case study 1: Burgers map as CPRNG/controlled system with CF_Simple_,
- case study 2: Burgers map as CPRNG/controlled system with CF_UNI_,
- case study 3: delayed logistic map as CPRNG/controlled system with CF_Simple_,
- case study 4: delayed logistic map as CPRNG/controlled system with CF_UNI_,
- case study 5: Lozi map as CPRNG/controlled system with CF_Simple_,
- case study 6: Lozi map as CPRNG/controlled system with CF_UNI_.

This work is focused on the utilization of the chaos driven DE for tuning of parameters for ETDAS control method to stabilize desired unstable periodic orbits (UPO). In the described research, desired UPO was p-1 (stable state). The original control method, ETDAS, in the discrete form suitable for discrete chaotic maps has the following form:

(8) $$\begin{array}{c} F_{n}=K\left[\left(1-R\right)S_{n-m}-x_{n}\right],\\ S_{n}=x_{n}+RS_{n-m}, \end{array}$$

where *K* and *R* are adjustable constants, *F* is the perturbation, *S* is given by a delay equation utilizing previous states of the system, and *m* is the period of *m*-periodic orbit to be stabilized. The perturbation *F*_*n*_ in (8) may have arbitrarily large value, which can cause the divergence of the system. Therefore, *F*_*n*_ should have a value between—*F*_max⁡_ and *F*_max⁡_. The ranges of all evolutionary estimated parameters are given in Table 1.

Within the research, a total number of 50 simulations with ChaosDE were carried out for each case study. The parameter settings for ChaosDE were obtained analytically based on numerous experiments and simulations (see Table 2). Experiments were performed in an environment of* Wolfram Mathematica*; PRNG operations therefore used the built-in* Mathematica software* pseudorandom number generator. All experiments used different initialization; that is, different initial population was generated in each run of chaos driven DE.

## 7. Results

All simulations were successful and have given new optimal settings for ETDAS control method securing the fast stabilization of the chaotic system at required behaviour (p-1 orbit). Tables 4, 5, 6, 7, 8, and 9 contain the simple statistical overview of optimization/simulation results as well as the best founded individual solutions of parameters setup for ETDAS control method, corresponding final CF value, also, the Istab. Value representing the number of iterations required for stabilization on desired UPO, and further the average error between desired output value and real system output from the last 20 iterations.

**Figure 11:**
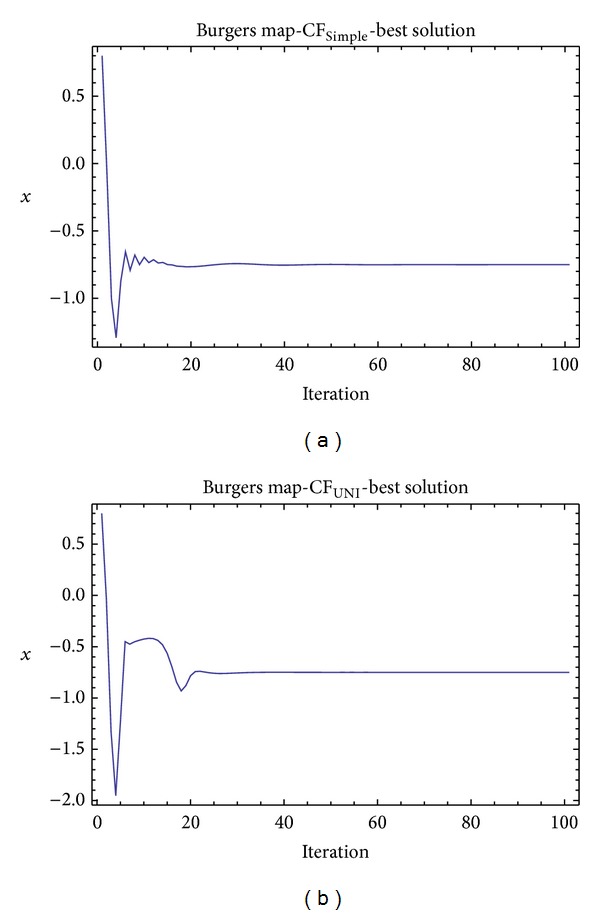
Simulation of the best individual solution, ChaosDE and Burgers map: case study 1,CF_Simple_ (a); case study 2, CF_UNI_ (b).

**Figure 12:**
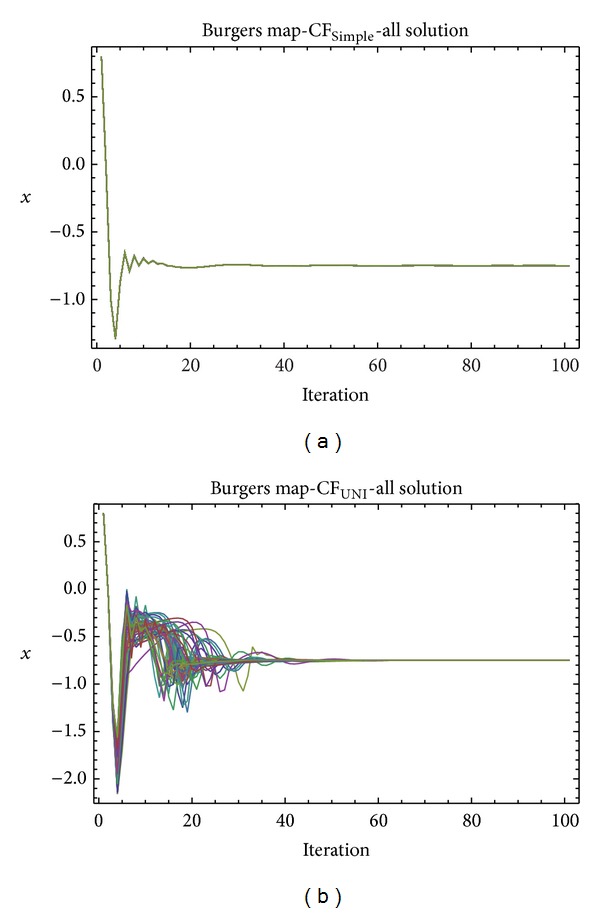
Simulation of all the 50 independent runs of EA, ChaosDE and Burgers map: case study 2, CF_Simple_ (a); case study 1, CF_UNI_ (b).

**Figure 13:**
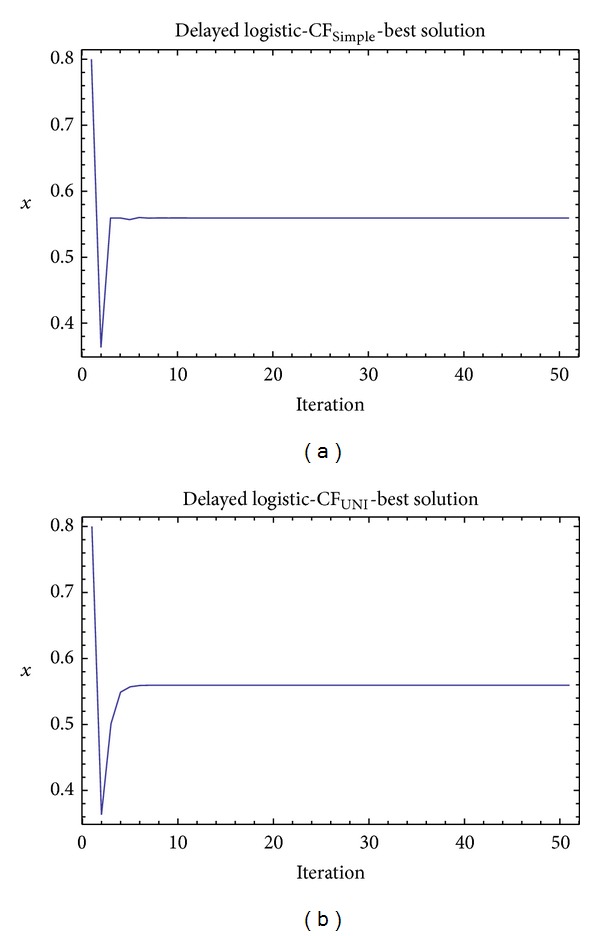
Simulation of the best individual solution, ChaosDE and delayed logistic: case study 3, CF_Simple_ (a); case study 4, CF_UNI_ (b).

**Figure 14:**
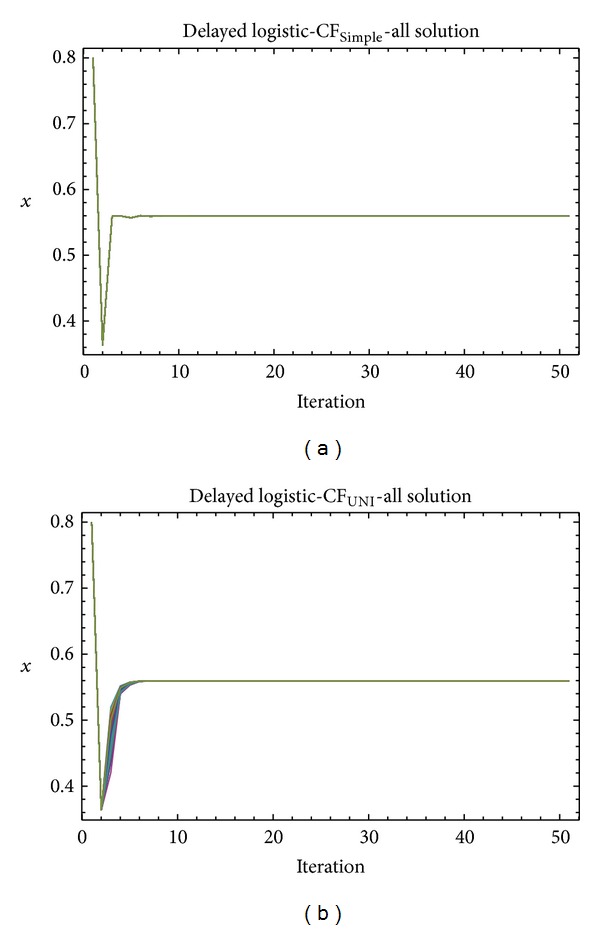
Simulation of all the 50 independent runs of EA, ChaosDE and Delayed logistic: case study 3, CF_Simple_ (a); case study 4, CF_UNI_ (b).

**Figure 15:**
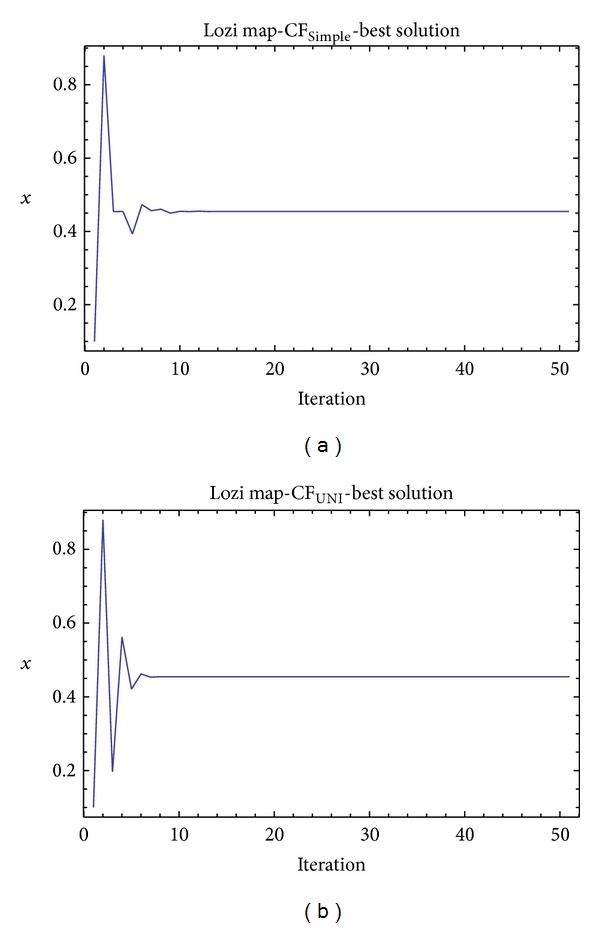
Simulation of the best individual solution, ChaosDE and Lozi map: case study 5, CF_Simple_ (a); case study 6, CF_UNI_ (b).

**Figure 16:**
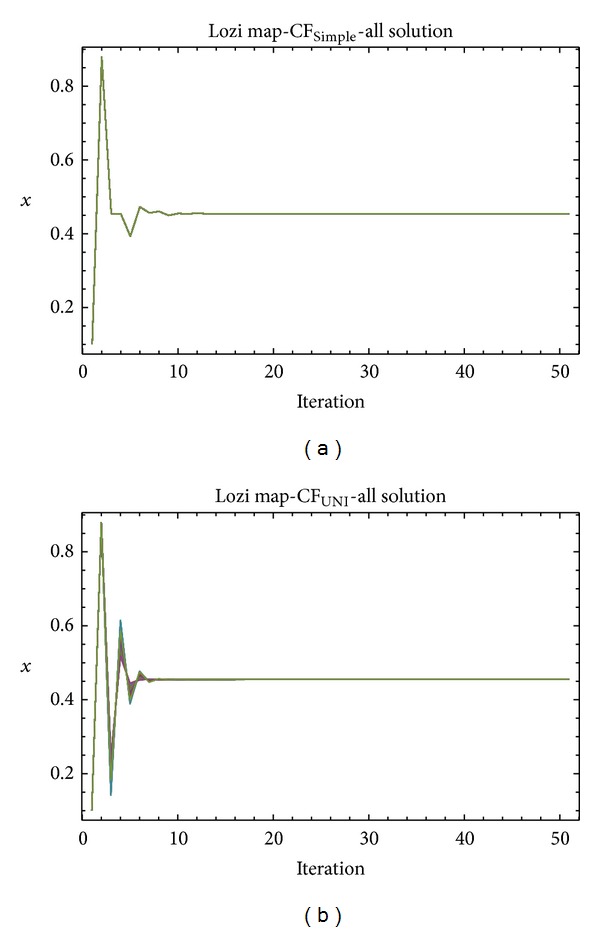
Simulation of the all 50 independent runs of EA, ChaosDE and Lozi map: case study 5, CF_Simple_ (a); case study 6, CF_UNI_ (b).

Graphical simulation outputs of the best individual solutions for particular case studies are depicted in Figures 11, 13, and 15, whereas Figures 12, 14, and 16 show the simulation output of all 50 runs of ChaosDE, thus confirming the robustness of this approach. For the illustrative purposes, all graphical simulations outputs are depicted only for the variable *x* of the chaotic systems.

The values for desired p-1 UPOs (fixed points) of unperturbed chaotic systems based on the mathematical analysis of the systems are given in Table 3.

From the results presented in the Tables 4–9, it follows that the CF-simple is very convenient for evolutionary process, which means that repeated runs of EA are giving identical optimal results (i.e., very close to the possible global extreme). This is graphically confirmed in Figures 12, 14, and 16, which show all 50 simulations. All the runs are basically merged into one line.

On the other hand, the disadvantage of including of initial chaotic transient behavior of not stabilized system into the cost function value and subsequent resulting very tiny change of control method setting for extremely sensitive chaotic system is causing suppression of stabilization speed and numerical precision.

Results obtained in the cases utilizing the CF_UNI_ lend weight to the argument that the technique of pure searching for periodic orbits is advantageous for faster and more precise stabilization of chaotic system.

## 8. Conclusions

Based on obtained results, it may be claimed that the presented ChaosDE driven by selected discrete dissipative chaotic systems has given satisfactory results in the chaos control optimization issue.

The results show that the embedding of the chaotic dynamics in the form of chaotic pseudorandom number generator into the differential evolution algorithm may help to improve the performance and robustness of the DE. ChaosDE is able to obtain optimal solutions securing the very fast and precise stabilization for both convenient CF surface, in case of the CF-simple, as well as for the very chaotic and nonlinear CF surface in case of the CF-universal.

When comparing both the CF designs, the CF-simple is very convenient for evolutionary process (i.e., repeated runs are giving identical optimal results), but it has many limitations.

The second universal CF design brings the possibility of using it problem free for any desired behavior of arbitrary chaotic systems but at the cost of the highly chaotic CF surface. Nevertheless, the embedding of the chaotic dynamics into the evolutionary algorithms helped to deal with such an issue.

The primary aim of this work was not to develop any new pseudorandom number generator, which should normally pass many statistical tests but to show that through embedding the hidden chaotic dynamics into the evolutionary process in the form of chaotic pseudorandom number generators may help to obtain better results and avoid problems connected with evolutionary computation such as premature convergence and stagnation in local extremes.

Future plans include testing of different chaotic systems, either manually or evolutionary tuning of parameters of chaotic maps, further complex comparisons with different heuristics and obtaining a large number of results to perform statistical tests.

## Figures and Tables

**Table 1:** Estimated parameters.

Parameter	Min	Max
*K*	−2	2
*R*	0	0.99
*F*_max⁡_	0	0.9

**Table 2:** DE settings.

Parameter	Value
Population size	25
*F*	0.8
Cr	0.8
Generations	250
Max cost function evaluations (CFE)	6250

**Table 3:** The values for p-1 UPO.

Chaotic system	Values of p-1 UPO of unperturbed system
Burgers map	*x*_*F*_ = −0.7499
Delayed logistic map	*x*_*F*_ = 0.559471
Lozi map	*x*_*F*_ = 0.454545

**Table 4:** Simple CF statistics: joined case studies 1 and 2, Burgers map as CPRNG/controlled system.

Statistical data	Case study 1 CF_simple_	Case study 2 CF_UNI_
	CF value	CF value
Min	2.16199	1.05 *·* 10^−6^
Max	2.16199	0.0103
Average	2.16199	6.67 *·* 10^−4^
Median	2.16199	5.32 *·* 10^−7^
Std. dev.	5.58 *·* 10^−11^	1.89 *·* 10^−3^
Average full stab. (iteration)	45	35

**Table 5:** Characteristics of the best solution: joined case studies 1 and 2, Burgers map as CPRNG/controlled system.

Parameter	Case study 1 CF_simple_	Case study 2 CF_UNI_
	Value	Value
*K*	1.22847	0.732498
*F*_max⁡_	0.9	0.48495
*R*	0.574997	0.811742
CF value	2.16199	1.05 *·* 10^−6^
Istab. Value	45	25
Average error per iteration	5.86 *·* 10^−5^	1.21 *·* 10^−8^

**Table 6:** Simple CF statistics: joined case studies 3 and 4, delayed logistic as CPRNG/controlled system.

Statistical data	Case study 3 CF_simple_	Case study 4 CF_UNI_
	CF value	CF value
Min	0.199798	2.3 *·* 10^−15^
Max	0.199798	2.7 *·* 10^−15^
Average	0.199798	2.44222 *·* 10^−15^
Median	0.199798	2.40551 *·* 10^−15^
Std. dev.	5.11 *·* 10^−16^	9.04629 *·* 10^−17^
Average full stab. (iteration)	10	7.5

**Table 7:** Characteristics of the best solution: joined case studies 3 and 4, delayed logistic as CPRNG/controlled system.

Parameter	Case study 3 CF_simple_	Case study 4 CF_UNI_
	Value	Value
*K*	1.29837	1.31355
*F*_max⁡_	0.394579	0.336294
*R*	0.01	0.010219
CF value	0.199798	2.3 *·* 10^−15^
Istab. Value	10	8
Average error per iteration	2.22 *·* 10^−17^	0

**Table 8:** Simple CF statistics: joined case studies 5 and 6, Lozi map as CPRNG/controlled system.

Statistical data	Case study 5 CF_simple_	Case study 6 CF_UNI_
	CF value	CF value
Min	0.520639	3.5331 *·* 10^−15^
Max	0.520639	4.0551 *·* 10^−15^
Average	0.520639	3.8063 *·* 10^−15^
Median	0.520639	3.6352 *·* 10^−16^
Std. dev.	2.41 *·* 10^−15^	1.19 *·* 10^−16^
Average full stab. (iteration)	32	11

**Table 9:** Characteristics of the best solution: joined case studies 5 and 6, Lozi map as CPRNG/controlled system.

Parameter	Case study 5 CF_simple_	Case study 6 CF_UNI_
	Value	Value
*K*	−1.11259	−0.859989
*F*_max⁡_	0.9	0.643099
*R*	0.289232	0.065669
CF value	0.520639	3.5331 *·* 10^−15^
Istab. Value	21	9
Average error per iteration	7.21 *·* 10^−15^	0

## References

[1] Ott E, Grebogi C, Yorke JA (1990). Controlling chaos. *Physical Review Letters*.

[2] Zelinka I (2009). Real-time deterministic chaos control by means of selected evolutionary techniques. *Engineering Applications of Artificial Intelligence*.

[3] Zelinka I, Senkerik R, Navratil E (2009). Investigation on evolutionary optimization of chaos control. *Chaos, Solitons and Fractals*.

[4] Senkerik R, Zelinka I, Davendra D, Oplatkova Z (2010). Utilization of SOMA and differential evolution for robust stabilization of chaotic Logistic equation. *Computers & Mathematics with Applications*.

[5] Pyragas K (1995). Control of chaos via extended delay feedback. *Physics Letters A*.

[6] Just W, Schuster HG (1999). Principles of time delayed feedback control. *Handbook of Chaos Control*.

[7] Senkerik R, Oplatkova Z, Zelinka I, Davendra D (2013). Synthesis of feedback controller for three selected chaotic systems by means of evolutionary techniques: analytic programming. *Mathematical and Computer Modelling*.

[8] Kominkova Oplatkova Z, Senkerik R, Zelinka I, Pluhacek M (2013). Analytic programming in the task of evolutionary synthesis of a controller for high order oscillations stabilization of discrete chaotic systems. *Computers and Mathematics with Applications*.

[9] Pyragas K (1992). Continuous control of chaos by self-controlling feedback. *Physics Letters A*.

[10] Kennedy J, Eberhart R Particle swarm optimization.

[11] Abedini M, Vatankhah R, Assadian N (2012). Stabilizing chaotic system on periodic orbits using multi-interval and modern optimal control strategies. *Communications in Nonlinear Science and Numerical Simulation*.

[12] Sadeghpour M, Salarieh H, Vossoughi G, Alasty A (2011). Multi-variable control of chaos using PSO-based minimum entropy control. *Communications in Nonlinear Science and Numerical Simulation*.

[13] Coelho LDS, Grebogi RB (2010). Chaotic synchronization using PID control combined with population based incremental learning algorithm. *Expert Systems with Applications*.

[14] Shirazi MJ, Vatankhah R, Boroushaki M, Salarieh H, Alasty A (2012). Application of particle swarm optimization in chaos synchronization in noisy environment in presence of unknown parameter uncertainty. *Communications in Nonlinear Science and Numerical Simulation*.

[15] Zelinka I, Raidl A, Zelinka I, Celikovsky S, Richter H, Chen G (2010). Evolutionary synchronization of chaotic systems. *Evolutionary Algorithms and Chaotic Systems*.

[16] Richter H, Reinschke KJ (2000). Optimization of local control of chaos by an evolutionary algorithm. *Physica D: Nonlinear Phenomena*.

[17] Zelinka I, Chen G, Celikovsky S (2008). Chaos synthesis by means of evolutionary algorithms. *International Journal of Bifurcation and Chaos in Applied Sciences and Engineering*.

[18] Zelinka I, Chadli M, Davendra D, Senkerik R, Jasek R (2013). An investigation on evolutionary reconstruction of continuous chaotic systems. *Mathematical and Computer Modelling*.

[19] Aydin I, Karakose M, Akin E (2010). Chaotic-based hybrid negative selection algorithm and its applications in fault and anomaly detection. *Expert Systems with Applications*.

[20] Caponetto R, Fortuna L, Fazzino S, Xibilia MG (2003). Chaotic sequences to improve the performance of evolutionary algorithms. *IEEE Transactions on Evolutionary Computation*.

[21] Davendra D, Zelinka I, Senkerik R (2010). Chaos driven evolutionary algorithms for the task of PID control. *Computers and Mathematics with Applications*.

[22] Price KV, Storn RM, Lampinen JA (2005). *Differential Evolution—a Practical Approach to Global Optimization*.

[23] Zelinka I (2004). SOMA—self-organizing migrating algorithm. *New Optimization Techniques in Engineering*.

[24] Coelho LDS, Mariani VC (2009). A novel chaotic particle swarm optimization approach using Hénon map and implicit filtering local search for economic load dispatch. *Chaos, Solitons & Fractals*.

[25] Senkerik R, Davendra D, Zelinka I, Pluhacek M, Oplatkova Z Aninvestigation on the chaos driven differential evolution: an initial study.

[26] Davendra D, Zelinka I, Senkerik R, Bialic-Davendra M, Davendra D (2010). Chaos driven evolutionary algorithm for the traveling salesman problem. *Traveling Salesman Problem, Theory and Applications*.

[27] Davendra D, Bialic-Davendra M, Senkerik R Scheduling the lot-streaming flowshop scheduling problem with setup time with the chaos-induced enhanced differential evolution.

[28] Pluhacek M, Senkerik R, Davendra D, Kominkova Oplatkova Z, Zelinka I (2013). On the behavior and performance of chaos driven PSO algorithm with inertia weight. *Computers and Mathematics with Applications*.

[29] Pluhacek M, Senkerik R, Zelinka I, Davendra D Chaos PSO algorithm driven alternately by two different chaotic maps—an initial study.

[30] Pluhacek M, Senkerik R, Zelinka I, Herrero Á, Baruque B, Klett F Multiple choice strategy based PSO algorithm with chaotic decision making—a preliminary study.

[31] Coelho LDS, Mariani VC (2012). Firefly algorithm approach based on chaotic Tinkerbell map applied to multivariable PID controller tuning. *Computers and Mathematics with Applications*.

[32] Davendra D, Zelinka I, Celikovsky S, Richter H, Chen G (2010). Evolutionary algorithms and the edge of chaos. *Evolutionary Algorithms and Chaotic Systems*.

[33] Senkerik R, Pluhacek M, Zelinka I, Davendra D, Oplatkova Z, Jasek R, Zelinka I, Duy VH, Cha J (2014). Evolutionary control of chaotic Lozi map by means of chaos driven differential evolution. *AETA 2013: Recent Advances in Electrical Engineering and Related Sciences*.

[34] Senkerik R, Zelinka I, Pluhacek M, Kominkova Oplatkova Z (2014). Evolutionary control of chaotic burgers map by means of chaos enhanced differential evolution. *International Journal of Mathematics and Computers in Simulation*.

[35] Senkerik R, Pluhacek M, Zelinka I, Davendra D, Oplatkova Z, Jasek R Chaos driven differential evolution in the task of evolutionary control of delayed logistic chaotic system.

[36] Tasgetiren MF, Suganthan PN, Pan QK (2010). An ensemble of discrete differential evolution algorithms for solving the generalized traveling salesman problem. *Applied Mathematics and Computation*.

[37] Onwubolu GC, Davendra D (2009). *Differential Evolution: A handbook for Permutation-based Combinatorial Optimization*.

[38] Wang L, Qu H, Chen T, Yan FP (2013). An effective hybrid self-adapting differential evolution algorithm for the joint replenishment and location-inventory problem in a three-level supply chain. *The Scientific World Journal*.

[39] Das S, Abraham A, Chakraborty UK, Konar A (2009). Differential evolution using a neighborhood-based mutation operator. *IEEE Transactions on Evolutionary Computation*.

[40] Choi TJ, Ahn CW, An J (2013). An adaptive cauchy differential evolution algorithm for global numerical optimization. *The Scientific World Journal*.

[41] Qin AK, Huang VL, Suganthan PN (2009). Differential evolution algorithm with strategy adaptation for global numerical optimization. *IEEE Transactions on Evolutionary Computation*.

[42] Zhang J, Sanderson AC JADE: self-adaptive differential evolution with fast and reliable convergence performance.

[43] Zhang J, Sanderson AC Self-adaptive multi-objective differential evolution with direction information provided by archived inferior solutions.

[44] Senkerik R, Davendra D, Zelinka I, Oplatkova Z Chaos driven differential evolution in the task of chaos control optimization.

[45] Storn R, Price K (1997). Differential evolution—a simple and efficient heuristic for global optimization over continuous spaces. *Journal of Global Optimization*.

[46] Price KV, Corne D, Dorigo M, Glover F (1999). An introduction to differential evolution. *New Ideas in Optimization*.

[47] Sprott JC (2003). *Chaos and Time-Series Analysis*.

[48] Aziz-Alaoui MA, Robert C, Grebogi C (2001). Dynamics of a Hénon-Lozi-type map. *Chaos, Solitons & Fractals*.

